# High-resolution analysis of baculovirus-induced host manipulation in the domestic silkworm, *Bombyx mori*

**DOI:** 10.1017/S0031182020001924

**Published:** 2021-01

**Authors:** Hiroyuki Hikida, Susumu Katsuma

**Affiliations:** Department of Agricultural and Environmental Biology, Graduate School of Agricultural and Life Sciences, The University of Tokyo, 1-1-1, Yayoi, Bunkyo-ku, Tokyo 113-8657, Japan

**Keywords:** Baculovirus, *Bombyx mori*, host manipulation, time-lapse recording

## Abstract

Many parasites manipulate host behaviour to enhance their transmission. Baculoviruses induce enhanced locomotory activity (ELA) combined with subsequent climbing behaviour in lepidopteran larvae, which facilitates viral dispersal. However, the mechanisms underlying host manipulation system are largely unknown. Previously, larval locomotion during ELA was summarized as the distance travelled for a few minutes at several time points, which are unlikely to characterize ELA precisely, as ELA typically persists for several hours. In this study, we modified a recently developed method using time-lapse recording to characterize locomotion of *Bombyx mori* larvae infected with *B*. *mori* nucleopolyhedrovirus (BmNPV) for 24 h at 3 s resolution. Our data showed that the locomotion of the mock-infected larvae was restricted to a small area, whereas the BmNPV-infected larvae exhibited a large locomotory area. These results indicate that BmNPV dysregulates the locomotory pattern of host larvae. Furthermore, both the mock- and BmNPV-infected larvae showed periodic cycles of movement and stationary behaviour with a similar frequency, suggesting the physiological mechanisms that induce locomotion are unaffected by BmNPV infection. In contrast, the BmNPV-infected larvae exhibited fast and long-lasting locomotion compared with mock-infected larvae, which indicates that locomotory speed and duration are manipulated by BmNPV.

## Introduction

Manipulation of host behaviour is a strategy commonly used by parasites to enhance their proliferation in the environment (Moore, 2002). For example, hairworms cause crickets to jump into the water, where the hairworms can search for their mates (Thomas *et al*., 2002), and *Ophiocordyceps* fungi make ants to bite onto vegetation at an elevated position, which is thought to enable the fungi to disperse their spores (Andersen *et al*., 2009). Baculovirus is an insect-specific large DNA virus, which mainly infects lepidopteran larvae and some members of the virus induce dramatic alterations in the behaviour of lepidopteran hosts (Goulson, 1997). Baculovirus-infected larvae exhibit horizontal hyperactivity, which is called enhanced locomotory activity (ELA), followed by vertical movement, which is known as climbing behaviour (CB) or tree-top disease. These virus-induced host manipulations finally lead the larvae to the upper plant foliage, where they die. Baculoviruses encode cathepsin and chitinase in their genome, which cooperate to liquefy the larval body at a late stage of infection (Ohkawa *et al*., 1994; Hawtin *et al*., 1997). The combination of larval death at an elevated position, and liquefaction of their body is thought to facilitate viral dispersal with rainfall or avian predation (Entwistle *et al*., 1993; D'Amico and Elkinton, 1995).

Baculovirus-induced host manipulation was first quantified using *Mamestra brassicae* multiple nucleopolyhedrovirus (MbMNPV) and *M. brassicae* larvae, which revealed that MbMNPV-infected larvae exhibit a higher degree of horizontal dispersal and die at an elevated position (Vasconcelos *et al*., 1996; Goulson, 1997). Recent studies identified the baculovirus-encoding genes *protein tyrosine phosphatase* (*ptp*) and *ecdysteroid UDP-glucosyltransferase* (*egt*) as factors for inducing ELA and CB, respectively (Kamita *et al*., 2005; Hoover *et al*., 2011). The deletion of *ptp* in *Bombyx mori* nucleopolyhedrovirus (BmNPV) and *Autographa californica* multiple nucleopolyhedrovirus (AcMNPV) impairs the horizontal locomotory activity of infected **B*. mori* and *Spodoptera exigua* larvae, respectively (Kamita *et al*., 2005; Katsuma *et al*., 2012; van Houte *et al*., 2012). As the *ptp* deletion affects a degree of horizontal dispersal but not climbing height during AcMNPV infection, ELA and CB are thought to be governed by different mechanisms (van Houte *et al*., 2014*a*). The deletion of *egt* in *Lymantria dispar* multiple nucleopolyhedrovirus and *Spodoptera exigua* multiple nucleopolyhedrovirus mitigates the climbing height of *L. dispar* (Hoover *et al*., 2011) and *S. exigua* larvae (Han *et al*., 2015), respectively, although the deletion of *egt* does not alter host behaviour in BmNPV- or AcMNPV-infected larvae (Katsuma and Shimada, 2015; Ros *et al*., 2015).

In these previous studies, larval locomotory activity was assessed using sampling points typically involving brief observations at intervals of several hours. As ELA and CB are events that continue for hours or days, a more precise method was required to continuously evaluate larval locomotory activity during baculovirus infection in order to elucidate the mechanisms underlying baculovirus manipulation of host behaviour. Recently, we developed a novel method to continuously observe the locomotion of BmNPV-infected *B. mori* larvae using time-lapse recording at 20 s resolution for 24 h, followed by a video analysis to trace larval locomotion (Hikida *et al*., 2020). In this study, the method was further developed and horizontal locomotion of the BmNPV- and mock-infected larvae was evaluated at 3 s resolution. Our results identified host properties that were altered in response to BmNPV infection.

## Materials and methods

### Insects, cell lines and viruses

*Bombyx mori* larvae (Kinshu × Showa) were reared on artificial diet as previously described (Nakanishi *et al*., 2010). BmN-4 cells were cultured in TC-100 medium supplemented with 10% fetal bovine serum at 26°C. The T3 strain was used as the wild-type BmNPV (Maeda *et al*., 1985) and propagated in BmN-4 cells. Viral titre was determined by a plaque assay method with BmN-4 cells.

### Locomotion assays

**Fig. 1.**
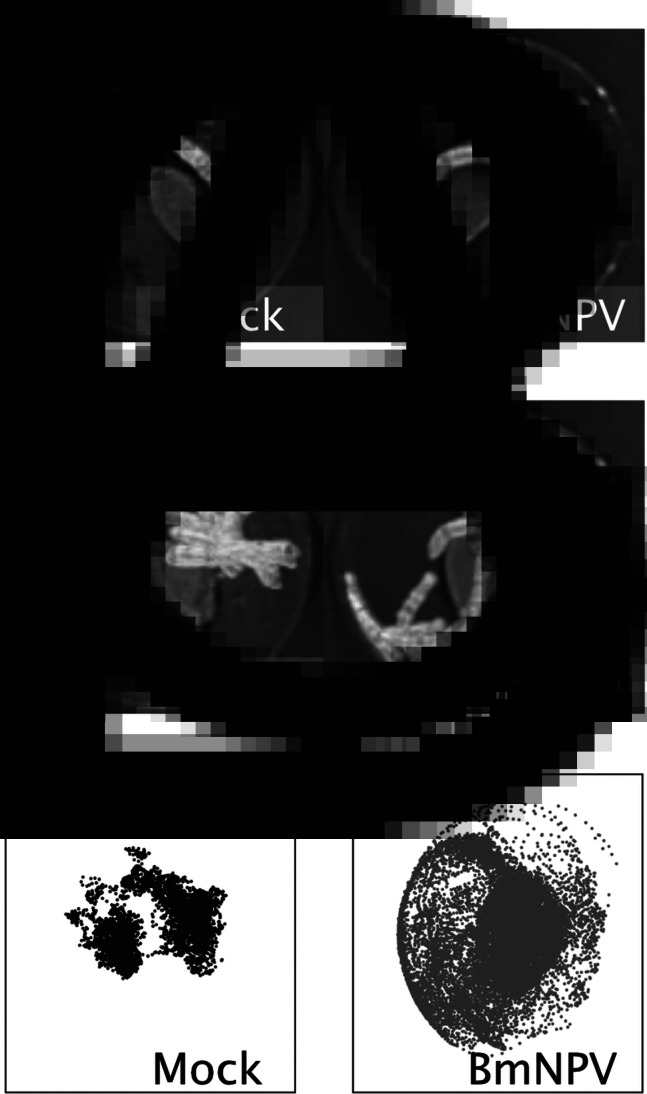
(A) Representative image of the larvae. A portion of artificial diet was placed at the centre of the dish. (B) An image generated from overlapping frames from the video. (C) The distribution of larvae during each 24 h observation was plotted. Data from each individual larva are presented in Fig. S1.

Locomotion assays were performed as previously described (Hikida *et al*., 2020). Fourth-instar larvae were starved for several hours and then injected intrahaemocoelically with a viral suspension containing 10^5^ plaque-forming units of budded virus or a control medium with 5 mg mL^−1^ kanamycin (Wako). Injected larvae were reared in an incubator at 25°C and 16 h:8 h L:D photoperiod until the observations started. At 72 h post-infection, each larva (*n* = 6) was placed in a 100 mm cell culture dish. A portion of artificial diet was provided, the amount of which was sufficient to feed the larvae during the observation. A piece of black paper was placed at the bottom of each dish. Locomotion was recorded at 3 s intervals using a time-lapse camera (TLC200-pro, Bruno) under light conditions for 24 h. The experiments were performed twice independently, hereafter designated as experiment #1 and #2. A rectangle of 520 × 400 pixels was cropped from an original video. The cropped video was converted to greyscale (Fig. 1A), and a background image was created as the average intensity. The background was subtracted from all frames in the greyscale video and then subjected to binarization. The processed video was used to determine the larval positions in each frame by calculating the centre of the mass of detected particles. These image-processing steps were conducted using the Image J software (Schneider *et al*., 2012).

### Data analysis

Data including X- and Y-coordinates of the centre of mass were analysed using custom Python scripts. First, the coordinates were plotted onto the original video and were checked to confirm the appropriate larval positions. Inappropriate points (i.e. dots outside the dish) identified in this analysis were removed from the dataset. We found that the positions of some of the mock-infected larvae were mistakenly mapped because these larvae were so inactive that the method was unable to create an appropriate background image. We excluded these mock-infected larvae (one larva per experiment) from further analysis. The adjusted data were utilized to calculate the larval travel distance as pixels between two adjacent frames. When multiple points were assigned to a single larva in a single frame, we used the average of the coordinates as the larval position. In case in which a larval position was missing in a frame, its position in the immediately previous frame was used for the calculations.

### Quantification of larval locomotion

The total travel distance was calculated as the sum of travel distance between two adjacent frames for 24 h. When the distance of the larval position between two adjacent frames exceeded 0.5 pixels, we scored larval locomotion as ‘movement’; otherwise, locomotion was scored as ‘stationary behaviour’. Locomotory speed was calculated as the distance travelled for 3 s when the larvae were moving. The average travel distance for each 30 s period was further calculated and when it exceeded 0.5 pixels, the larva was assumed to continue moving during the 30 s period. The sequential 30 s periods in which the larvae kept moving were defined as a single continuous locomotion. Locomotory duration was calculated as the number of 30 s periods consisting of a single continuous locomotion. Locomotory frequency was also counted as the number of single continuous locomotion events throughout the observation.

### Statistical analysis

A rank-sum test was employed to compare the total travel distance, median locomotory duration, frequency of locomotion and median travel distance during the larval movement between the mock- and BmNPV-infected larvae.

## Results

### Tracing larval locomotion during 24 h observation

**Fig. 2.**
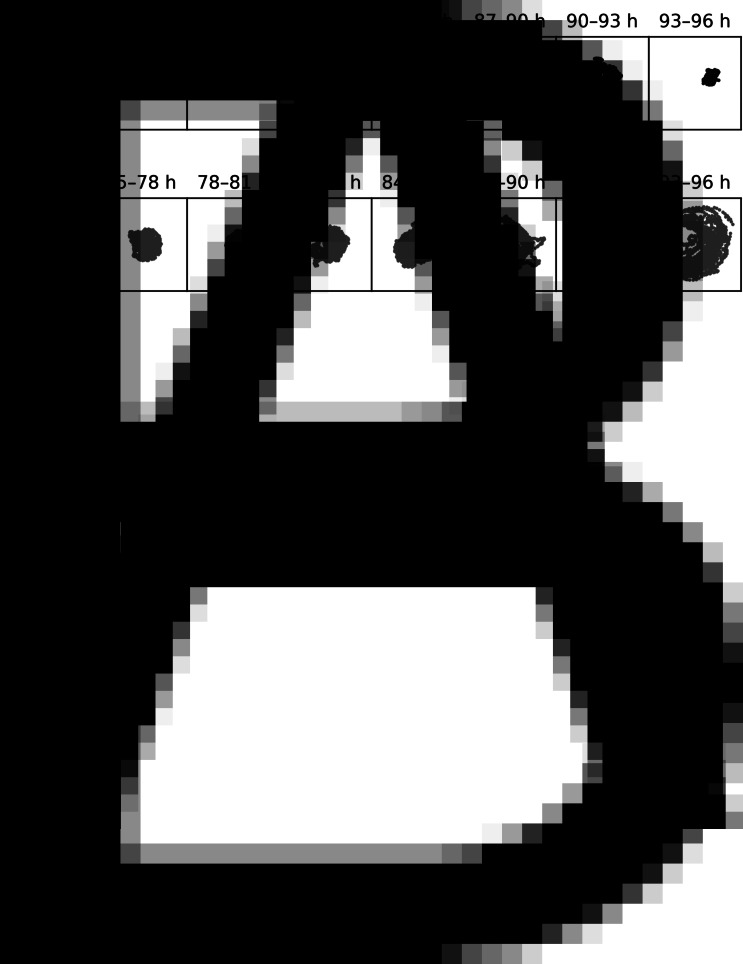
The distribution of individual larvae in 3 h segments. The time range is shown above each of the boxes. Each dot indicates the larval position in a frame. A representative larva was selected from the mock-infected larvae (A) and BmNPV-infected larvae (B) in experiment #1. Data from each individual larva are presented in Fig. S2. Dark and light colours indicate the mock- and BmNPV-infected larvae, respectively.

Each mock- or BmNPV-infected larva was placed in a cell culture dish with a portion of artificial diet and its locomotion was recorded for 24 h from 72 hpi when typical ELA was not observed (Fig. 1A and Supplementary video). No larvae moulted to 5^th^ instar and most of the infected larvae were alive during the observation. All the infected larvae died after the experiment. Generally, the locomotory area of BmNPV-infected larvae covered a larger area of the dish compared with the mock-infected ones (Fig. 1B and Supplementary video). The positions of each larva during a 24 h observation were plotted in a single image, and the locomotory pattern of the mock- and BmNPV-infected larvae were compared. The mock-infected larvae remained close to the portion of artificial diet and exhibited little locomotion, whereas the locomotory area of the BmNPV-infected larvae covered almost the entire area of the dish in both experiments (Figs 1C and S1). To examine temporal changes in larval locomotory area, 24 h data were divided into eight 3 h segments and analysed separately. In all the 3 h segments, the mock-infected larvae remained close to a portion of artificial diet (Figs 2A and S2). The BmNPV-infected larvae exhibited food-oriented distribution with slightly larger distribution during the early time segments (Figs 2B and S2). However, this locomotory pattern was markedly altered in the later time segments as the BmNPV-infected larvae moved to and along the edge of the dish (Figs 2B and S2). In these later segments, few of the BmNPV-infected larvae remained at the centre of the dish.

### Larval locomotion with 3 s resolution

**Fig. 3.**
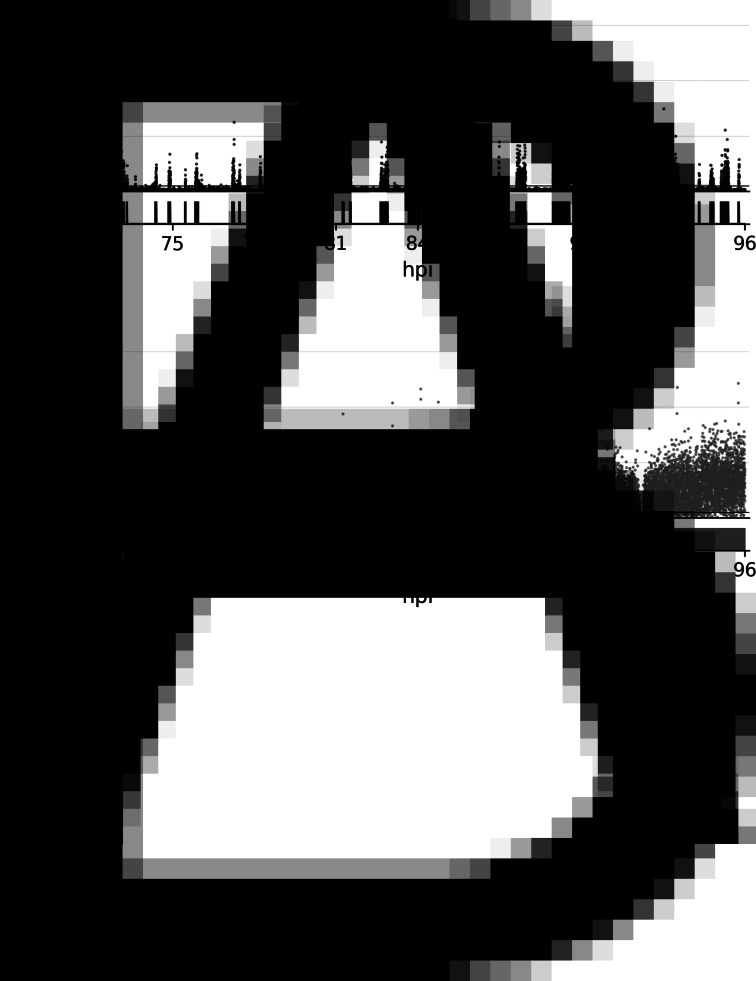
Travel distances between two adjacent frames, corresponding to 3 s intervals of observation. A representative larva was selected from the mock-infected larvae (A) and BmNPV-infected larvae (B) in experiment #1. The larvae are identical to those presented in Fig. 1. Black lines indicate the thresholds for movement and stationary behaviour. The rectangular diagrams below each of the scatter plots indicate duration of each single continuous locomotion. Data from each individual larva are presented in Fig. S3.

**Fig. 4.**
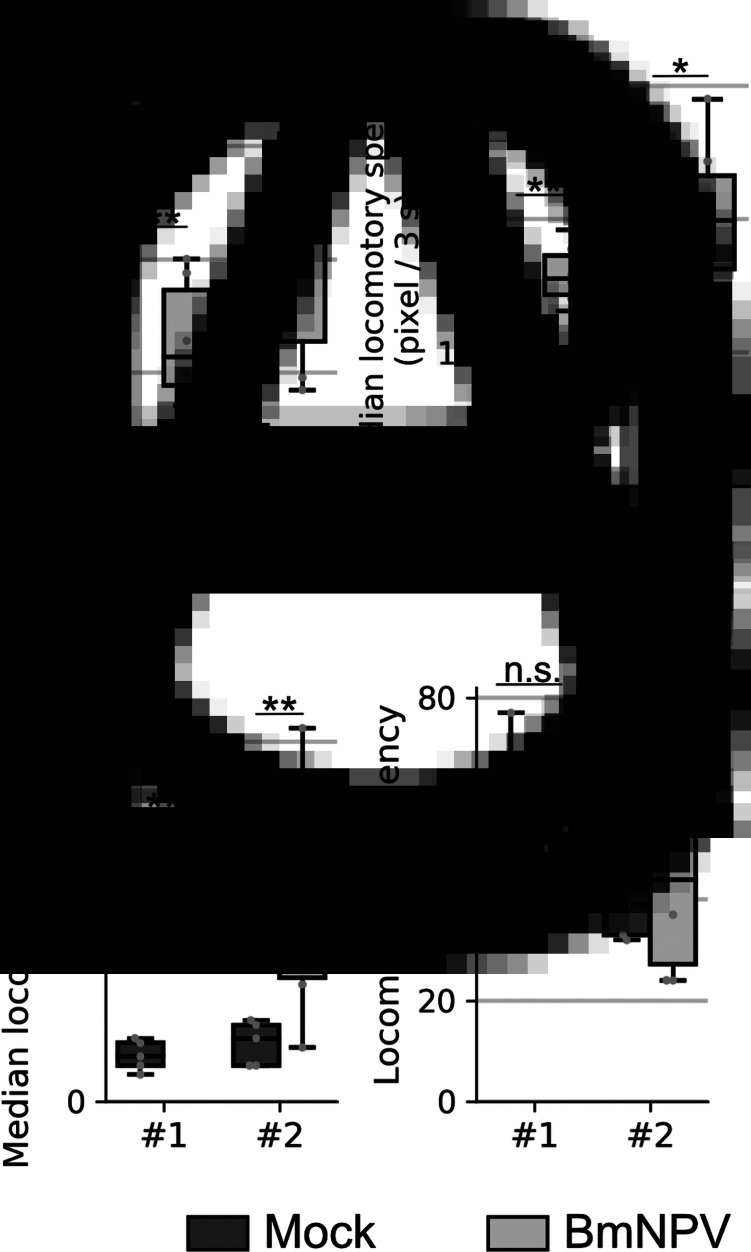
(A) The total travel distance over 24 h period shown as a box-and-whisker plot. (B) Median travel distance between two adjacent frames during larval movement. (C) Median locomotory duration. (D) Frequency of locomotion as the number of continuous locomotion events over 24 h period. Left and right boxes indicate the mock- and BmNPV-infected larvae, respectively. The number of experiments is indicated below the graphs. All boxes and whiskers show the quartiles and 1.5 interquartile range, respectively. Dots indicate values determined for each larva. *P < 0.05 and **P < 0.01 by rank-sum test, respectively.

The locomotion of the mock- and BmNPV-infected larvae was examined at 3 s resolution. Throughout the observation, the mock-infected larvae periodically exhibited movement and stationary behaviour (Figs. 3A and S3). The BmNPV-infected larvae also exhibited periodic cycles of movement and stationary behaviour but they moved more than the mock-infected larvae (Figs 3B and S3). As a consequence, the total travel distance of the BmNPV-infected larvae over 24 h was significantly greater than that of the mock-infected larvae (Fig. 4A). In order to identify further differences between the mock- and BmNPV-infected larvae, locomotory speed, duration and frequency were quantitatively evaluated. The median locomotory speeds during movement were significantly higher in the BmNPV-infected larvae than in the mock-infected larvae (Fig. 4B). Also, the median locomotory duration of the BmNPV-infected larvae was significantly longer than that of the mock-infected larvae (Fig. 4C). In contrast, no significant differences between the mock- and BmNPV-infected larvae were detected with respect to the frequencies of locomotion over a 24 h period (Fig. 4D).

## Discussion

Previous studies observed the locomotion of baculovirus-infected larvae at intervals of several hours, so information provided on how larval locomotion is altered during infection was limited (Kokusho *et al*., 2011, 2015; Katsuma *et al*., 2012; Katsuma and Shimada, 2015). This study revealed that infected larvae moved over a large area compared with mock-infected larvae (Fig. 1B and C). The segmented analysis revealed that both the mock- and BmNPV-infected larvae remained close to a portion of artificial diet at the early stages of infection. In contrast, BmNPV-infected larvae subsequently moved around the peripheral area of the dish and were rarely found at the centre at late stages of infection, whereas mock-infected larvae remained primarily at the centre of the dish (Fig. 2A and B). These results indicate that BmNPV infection disrupts the intrinsic larval locomotory patterns at a certain time point during the late stage of infection. Recent studies revealed that vertical movement of baculovirus-infected larvae is evoked by phototaxis and that a light signal perceived just before the onset of the movement fixes the larval locomotory direction during the very late stage of infection (van Houte *et al*., 2014*b*; Han *et al*., 2018). The results of our study suggest that the dysregulation of the host locomotory patterns might induce abnormal phototaxis in the infected larvae which is not observed in healthy larvae. As we did not evaluate the vertical movement or phototaxis of the infected *B. mori* larvae, further analysis will be necessary to determine whether the dysregulation of host locomotory patterns and the response to light signals are sequential events or whether they occur simultaneously.

The present method has enabled us to evaluate larval locomotory activity at 3 s resolution and to quantify the locomotory speed, duration and frequency, properties which have not been examined previously. As described previously, the mock-infected larvae showed periodic cycles of movement and stationary behaviour (Fig. 3A) (Nagata and Nagasawa, 2006; Nagata *et al*., 2011*a*; Kômoto, 2017). We found that the BmNPV-infected larvae also exhibited periodical locomotion with a similar frequency to those observed among the mock-infected larvae (Figs 3B and 4D). These results suggest that the physiological mechanisms that induce locomotion were unaffected by BmNPV infection. In contrast, locomotory speed and duration were significantly greater and longer, respectively, in the BmNPV-infected larvae compared with the mock-infected larvae, which resulted in longer travel distances (Fig. 4A–C). Furthermore, the BmNPV-infected larvae exhibited faster and long-lasting locomotion prior to the onset of dysregulation of the locomotory pattern, which resulted in larvae covering a slightly larger area than mock-infected ones at the earlier stages of observation (Figs 2 and 3). These findings are similar to those described previously when *B. mori* larvae were injected with HemaP, a small peptide which facilitates feeding behaviour. The HemaP-injected *B. mori* larvae moved over a slightly larger area compared with control larvae (Nagata *et al*., 2011*a*). *Bombyx mori* larvae harbour various peptides that regulate feeding behaviour. Some of these peptides are synthesized or recognized in the brain (Nagata *et al*., 2009, 2011*a*, 2011*b*, 2012). Previous studies revealed that BmNPV infection in the brain is crucial for the induction of host manipulation (Katsuma *et al*., 2012; Kokusho *et al*., 2015). Taken together, this suggests that BmNPV infection in the brain disrupts the expression and/or recognition of feeding-regulating peptides, which in turn might result in enhanced locomotory speed and duration.

This study revealed that BmNPV infection first enhances the speed and duration of host larval locomotion and then disrupts host locomotory patterns, inducing distinct larval behaviour. This is the first report, to our knowledge, to demonstrate the dysregulation of host locomotory patterns and the enhancement of larval locomotory speed and duration in response to baculovirus infection. As *B. mori* used in this study is a highly domesticated insect that exhibits distinct behavioural patterns compared with its putative ancestor, *B. mandarina* (Kômoto, 2017), additional studies will be required to determine if the results obtained here are applicable to other host–baculovirus combinations. It is also noted that the present observation was performed under constant light and the photoperiod was not controlled. The activity of *B. mori* larvae under dark conditions and the effect of photoperiod on behavioural alteration will be examined in future studies. Nevertheless, this study provides novel insights into the modification of larval behaviour during baculovirus infection, which may prove useful in deciphering the mechanisms underlying baculovirus-induced host behavioural manipulation.

## Supplementary material

For supplementary material accompanying this paper visit https://doi.org/10.1017/S0031182020001924. click here to view supplementary material
