# High-resolution analysis of baculovirus-induced host manipulation in the domestic silkworm, *Bombyx mori* – CORRIGENDUM

**DOI:** 10.1017/S0031182020002309

**Published:** 2021-04

**Authors:** Hiroyuki Hikida, Susumu Katsuma

**Keywords:** Baculovirus, Bombyx mori, host manipulation, time-lapse recording, corrigendum

The authors apologise for errors that appeared in the published article. The errors were in the format of some names of viruses and organisms within the Abstract, the second paragraph of the Introduction, and in the Materials and methods section of the article. The corrected text for these are given below:

## Abstract

Many parasites manipulate host behaviour to enhance their transmission. Baculoviruses induce enhanced locomotory activity (ELA) combined with subsequent climbing behaviour in lepidopteran larvae, which facilitates viral dispersal. However, the mechanisms underlying host manipulation system are largely unknown. Previously, larval locomotion during ELA was summarized as the distance travelled for a few minutes at several time points, which are unlikely to characterize ELA precisely, as ELA typically persists for several hours. In this study, we modified a recently developed method using time-lapse recording to characterize locomotion of *Bombyx mori* larvae infected with Bombyx mori nucleopolyhedrovirus (BmNPV) for 24 h at 3 s resolution.

Our data showed that the locomotion of the mock-infected larvae was restricted to a small area, whereas the BmNPV-infected larvae exhibited a large locomotory area. These results indicate that BmNPV dysregulates the locomotory pattern of host larvae. Furthermore, both the mock- and BmNPV-infected larvae showed periodic cycles of movement and stationary behavior with a similar frequency, suggesting the physiological mechanisms that induce locomotion

are unaffected by BmNPV infection. In contrast, the BmNPV-infected larvae exhibited fast and long-lasting locomotion compared with mock-infected larvae, which indicates that locomotory speed and duration are manipulated by BmNPV.

## **Introduction**, second paragraph:

Baculovirus-induced host manipulation was first quantified using Mamestra brassicae multiple nucleopolyhedrovirus (MbMNPV) and *Mamestra brassicae* larvae, which revealed that MbMNPV-infected larvae exhibit a higher degree of horizontal dispersal and die at an elevated position (Vasconcelos et al., 1996; Goulson, 1997). Recent studies identified the baculovirus-encoding genes *protein tyrosine phosphatase (ptp)* and *ecdysteroid UDP-glucosyltransferase (egt)* as factors for inducing ELA and CB, respectively (Kamita et al., 2005; Hoover et al., 2011). The deletion of *ptp* in Bombyx mori nucleopolyhedrovirus (BmNPV) and Autographa californica multiple nucleopolyhedrovirus (AcMNPV) impairs the horizontal locomotory activity of infected *Bombyx mori* and *Spodoptera exigua* larvae, respectively (Kamita et al., 2005; Katsuma et al., 2012; van Houte et al., 2012). As the *ptp* deletion affects a degree of horizontal dispersal but not climbing height during AcMNPV infection, ELA and CB are thought to be governed by different mechanisms (van Houte et al. 2014a). The deletion of *egt* in Lymantria dispar multiple nucleopolyhedrovirus and Spodoptera exigua multiple nucleopolyhedrovirus mitigates the climbing height of *Lymantria dispar* (Hoover et al., 2011) and *S. exigua* larvae (Han et al., 2015), respectively, although the deletion of *egt* does not alter host behaviour in BmNPV- or AcMNPV-infected larvae (Katsuma and Shimada, 2015; Ros et al., 2015).

## Materials and methods

### Insects, cell lines and viruses

*B. mori* larvae (Kinshu × Showa) were reared on artificial diet as previously described (Nakanishi et al., 2010). BmN-4 cells were cultured in TC-100 medium supplemented with 10% fetal bovine serum at 26°C. The T3 strain was used as the wildtype BmNPV (Maeda et al., 1985) and propagated in BmN-4 cells. Viral titre was determined by a plaque assay method with BmN-4 cells.
